# Impact of ultrasound diagnosis for chronic pelvic pain

**DOI:** 10.1097/MD.0000000000017281

**Published:** 2019-09-27

**Authors:** Xiao-hui Wang, Jing-jun Xu, Guang Yang, Tian-you Xin

**Affiliations:** a Department of Ultrasound; b Department of Gynecology, First Affiliated Hospital of Jiamusi University, Jiamusi; c Department of Ultrasound, Wuxi No.2 People's Hospital, Wuxi, China.

**Keywords:** chronic pelvic pain, sensitivity, specificity, ultrasound

## Abstract

**Background:**

This study aims to assess the impact of ultrasound diagnosis in patients with chronic pelvic pain (CPP).

**Methods:**

We will carry out a comprehensive electronic search from the databases below: PUBMED, EMBASE, Cochrane Library, PSYCINFO, Web of Science, Cumulative Index to Nursing and Allied Health Literature, Allied and Complementary Medicine Database, Chinese Biomedical Literature Database, China National Knowledge Infrastructure, and WANGFANG databases from inception to July 1, 2019. The case-controlled studies focusing on impact of ultrasound diagnosis for patients CPP will be included in this study. Two authors will independently conduct all study selection, data collection, and risk of bias assessment. The risk of bias assessment will be assessed using Quality Assessment of Diagnostic Accuracy Studies tool. We will apply RevMan V.5.3 software and Stata V.12.0 software for data pooling and statistical analysis.

**Results:**

This study will present pooled effect estimates regarding the impact of ultrasound diagnosis for CPP by assessing sensitivity, specificity, positive likelihood ratio, negative likelihood ratio, and diagnostic odds ratio of ultrasound to determine the diagnostic accuracy of ultrasound diagnosis for CPP.

**Conclusion:**

This study will provide modest evidence for the diagnostic accuracy of ultrasound in patients with CPP.

**Systematic review registration:**

PROSPERO CRD42019142799.

## 1 Introduction

Constant or intermittent chronic pelvic pain (CPP) is a common complaint in women.^[1–3]^ It often manifests in the pelvic and lower abdominal regions for more than 6 months.^[4–7]^ CPP often accompanies dysmenorrhoea, dyspareunia, dyschesia, and dysuria.^[8–10]^ The annual prevalence of CPP is 38 out of every 1000 women aged 15 to 73 years, but only 20% to 25% of them visit the doctor.^[2,11]^ Thus, CPP is the most common complaint in the gynecology clinic, accounting for more than 20% of all outpatient visits.^[12,13]^

The clinical management of CPP depends on the patients’ diagnosis.^[14,15]^ Ultrasound has been reported to help diagnose patients with CPP accurately.^[16–21]^ However, no study has systematically assessed its impact for patients with CPP. Thus, in this study, we will systematically evaluate the diagnostic impact of ultrasound for patients with CPP.

## 2 Methods

### 2.1 Objective

This study will aim to evaluate the impact of ultrasound in the diagnosis of patients with CPP.

### 2.2 Inclusion criteria for study selection

#### 2.2.1 Type of studies

This study will include case-controlled studies that evaluating the impact of ultrasound diagnosis of patients with CPP. However, we will exclude any other studies, such as animal studies, non-clinical studies, and case studies.

#### 2.2.2 Type of participants

In this study, the reports of patients with laboratory examination-proven CPP will be included.

#### 2.2.3 Type of index test

Index test: ultrasound will be utilized to diagnose patients with CPP. However, CPP combined with other diagnostic test will not be included.

Reference test: patients with laboratory examination-proven CPP will be considered as comparators.

#### 2.2.4 Type of outcome measurements

The primary outcomes consist of sensitivity and specificity. The secondary outcomes comprise of positive likelihood ratio, negative likelihood ratio, and diagnostic odds ratio.

### 2.3 Data sources and search strategy

#### 2.3.1 Electronic searches

The following databases will be comprehensively searched from inception to July 1, 2019: PUBMED, EMBASE, Cochrane Library, PSYCINFO, Web of Science, Cumulative Index to Nursing and Allied Health Literature, Allied and Complementary Medicine Database, Chinese Biomedical Literature Database, China National Knowledge Infrastructure, and WANGFANG databases. All electronic databases will be retrieved without any language limitations. The detailed search strategy for PUBMED is presented in Table 1. In addition, similar search strategies will be adapted to any other electronic databases.

**Table 1:**
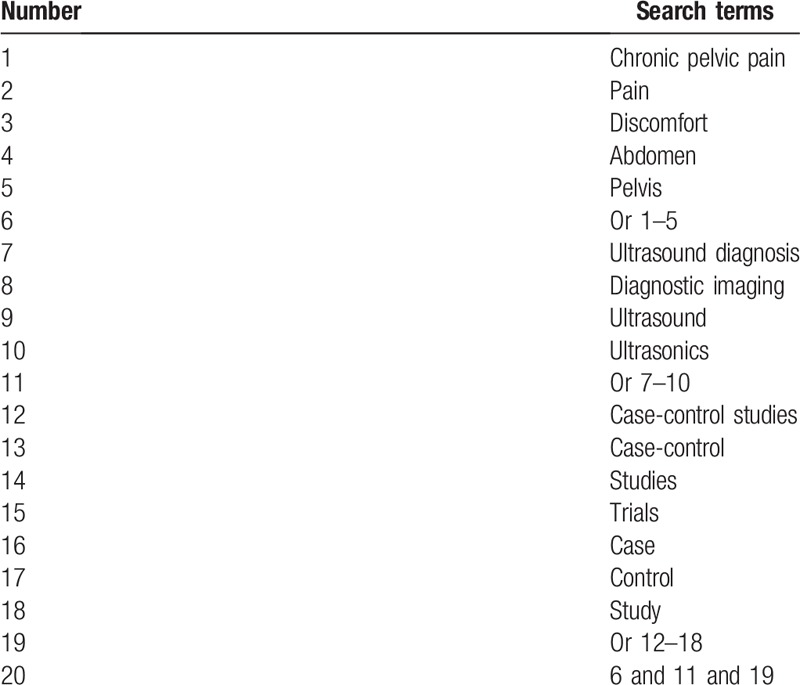
Search strategy utilized in PUBMED database.

#### 2.3.2 Other resources

We will also search conference proceedings, clinical registry, and reference lists of included studies.

### 2.4 Data collection and analysis

#### 2.4.1 Study selection

This study consists of two-stage screening process. First, the inclusion criteria will be performed by 2 independent authors to scan all titles and abstracts to check potential relevant studies. Studies will be rejected at first stage if they will not meet the eligibility criteria. Second, full literature copies of remaining studies will be reviewed to identify if they meet final selection. Any disagreements between 2 authors will be solved by consensus with the help of a third author. All study selection process will be detailed in a flow-diagram chart.

#### 2.4.2 Data extraction

Two authors will independently extract data from all eligible studies according to the data extraction spreadsheet. Any differences regarding the extracted variables between 2 authors will be solved by consensus with a third author. The data extraction consists of study characteristics, study design, patient characteristics, inclusion eligibility, study methods, index and reference tests, and outcome measurements. Any attempts will be tried to inquire missing information by contacting corresponding authors of all eligible studies.

### 2.5 Quality assessment

All quality assessments of included studies will be evaluated by methodological quality will be measured by Quality Assessment of Diagnostic Accuracy Studies tool.^[22]^ Two authors will independently assess the methodological quality of all included studies respectively. Any discrepancies between 2 authors will be solved by discussion with a third author.

### 2.6 Assessment of heterogeneity

Before data synthesis, we will assess the heterogeneity among eligible studies using *I*^*2*^ statistic. *I*^*2*^ ≤ 50% means low heterogeneity, while *I*^*2*^ > 50% indicates significant heterogeneity.

### 2.7 Statistical analysis

We will use RevMan V.5.3 software and Stata V.12.0 software for statistical analysis. Specific characteristics and findings of all included studies will be illustrated in tables. Each outcome will be calculated as descriptive statistics with 95% confidence intervals. In addition, a descriptive forest plot and a summary receiver operating characteristic plot will also be carried out.

If *I*^2^ ≤ 50%, we will use Mantel–Haenszel fixed-effects model to pool the data and meta-analysis will be conducted. Otherwise, Mantel–Haenszel random-effects model will be used to pool the data, subgroup analysis will be performed and meta-regression test will be operated to identify the sources of heterogeneity before the decision of whether outcome data should be synthesized. If there is still significant heterogeneity after subgroup analysis, we will only describe outcome results instead of data pooling and meta-analysis.

### 2.8 Subgroup analysis

This study will carry out subgroup analysis to identify any possible causes for significant heterogeneity based on the types of study characteristics, treatments, and controls.

### 2.9 Sensitivity analysis

We will perform sensitivity analysis to check stability of outcome results by eliminating the low methodological quality studies.

### 2.10 Reporting bias

For the assessment of reporting bias, we will produce funnel plots and associated regression tests to judge the publication bias among included studies.^[23]^

### 2.11 Ethics and dissemination

Publication in a relevant peer-reviewed journal and dissemination is at a peer-reviewed journal. This study will not need ethic approval, because no individual data are involved.

## 3 Discussion

No study has reported to assess the impact of ultrasound diagnosis of patients with CPP presently. This study firstly tries to systematically search and summarize the current relevant primary studies on the impact and to synthesize the effect estimates from all eligible studies. The results of this study will be likely to well inform the policy and decision making on diagnosis and healthcare for patients with CPP in the public or clinical practice.

## Author contributions

**Conceptualization:** Xiao-hui Wang, Tian-you Xin.

**Data curation:** Xiao-hui Wang, Jing-jun Xu, Guang Yang, Tian-you Xin.

**Formal analysis:** Xiao-hui Wang, Guang Yang.

**Funding acquisition:** Xiao-hui Wang.

**Investigation:** Tian-you Xin.

**Methodology:** Xiao-hui Wang, Jing-jun Xu, Guang Yang.

**Project administration:** Tian-you Xin.

**Resources:** Xiao-hui Wang, Jing-jun Xu.

**Software:** Xiao-hui Wang, Jing-jun Xu, Guang Yang, Tian-you Xin.

**Supervision:** Tian-you Xin.

**Validation:** Jing-jun Xu, Guang Yang, Tian-you Xin.

**Visualization:** Xiao-hui Wang, Jing-jun Xu.

**Writing – original draft:** Xiao-hui Wang, Jing-jun Xu, Guang Yang, Tian-you Xin.

**Writing – review & editing:** Xiao-hui Wang, Guang Yang, Tian-you Xin.

## References

[1] Stein SL Chronic pelvic pain. Gastroenterol Clin North Am 2013;42:785–800. 24280400 10.1016/j.gtc.2013.08.005

[2] Ahangari A Prevalence of chronic pelvic pain among women: an updated review. Pain Physician 2014;17:E141–7. 24658485

[3] Siedentopf F Sillem M Chronic pelvic pain in women. Schmerz 2014;28:300–4. 24903044 10.1007/s00482-014-1408-4

[4] Giamberardino MA Tana C Costantini R Pain thresholds in women with chronic pelvic pain. Curr Opin Obstet Gynecol 2014;26:253–9. 24921647 10.1097/GCO.0000000000000083

[5] Leong FC Complementary and alternative medications for chronic pelvic pain. Obstet Gynecol Clin North Am 2014;41:503–10. 25155128 10.1016/j.ogc.2014.05.001

[6] Ayorinde AA Macfarlane GJ Saraswat L Chronic pelvic pain in women: an epidemiological perspective. Womens Health (Lond) 2015;11:851–64. 26450216 10.2217/whe.15.30

[7] Siedentopf F Weijenborg P Engman M ISPOG European Consensus Statement - chronic pelvic pain in women (short version). J Psychosom Obstet Gynaecol 2015;36:161–70. 26514847 10.3109/0167482X.2015.1103732

[8] Speer LM Mushkbar S Erbele T Chronic pelvic pain in women. Am Fam Physician 2016;93:380–7. 26926975

[9] Bonnema R McNamara M Harsh J Primary care management of chronic pelvic pain in women. Cleve Clin J Med 2018;85:215–23. 29522389 10.3949/ccjm.85a.16038

[10] Daniels JP Khan KS Chronic pelvic pain in women. BMJ 2010;341:c4834. 20923840 10.1136/bmj.c4834

[11] Henzl MR Dysmenorrhoea; achievements and challenge. Sex Med Today 1985;9:7–11.

[12] Howard FM The role of laparoscopy in chronic pelvic pain: promise and pitfalls. Obstet Gynecol Surv 1993;48:357–87. 8327235 10.1097/00006254-199306000-00001

[13] Bedaiwy MA Allaire C Yong P Medical management of endometriosis in patients with chronic pelvic pain. Semin Reprod Med 2017;35:38–53. 28002850 10.1055/s-0036-1597308

[14] Carey ET Till SR As-Sanie S Pharmacological management of chronic pelvic pain in women. Drugs 2017;77:285–301. 28074359 10.1007/s40265-016-0687-8

[15] Till SR Wahl HN As-Sanie S The role of nonpharmacologic therapies in management of chronic pelvic pain: what to do when surgery fails. Curr Opin Obstet Gynecol 2017;29:231–9. 28604402 10.1097/GCO.0000000000000376

[16] Qin W Diagnostic value of transabdominal color Doppler ultrasound in chronic pelvic pain. J Clin Rationl Med 2018;11:111–2.

[17] Lin GT Reed-Maldonado AB Lin MF Effects and mechanisms of low-intensity pulsed ultrasound for chronic prostatitis and chronic pelvic pain syndrome. Int J Mol Sci 2016;17:10.3390/ijms17071057 PMC4964433 27376284

[18] Ozkan D Akkaya T Yildiz S Ultrasound-guided pulsed radiofrequency treatment of the pudendal nerve in chronic pelvic pain. Anaesthesist 2016;65:134–6. 26811947 10.1007/s00101-015-0133-4

[19] Dale WS Transvaginal ultrasound findings in women with chronic pelvic pain. Obstetr Gynecol 2000;95:S57.

[20] Okaro E Valentin L The role of ultrasound in the management of women with acute and chronic pelvic pain. Best Pract Res Clin Obstet Gynaecol 2004;18:105–23. 15123061 10.1016/j.bpobgyn.2003.09.012

[21] Okaro E Condous G Khalid A The use of ultrasound-based 'soft markers’ for the prediction of pelvic pathology in women with chronic pelvic pain--can we reduce the need for laparoscopy? BJOG 2006;113:251–6. 16487194 10.1111/j.1471-0528.2006.00849.x

[22] Whiting PF Rutjes AW Westwood ME QUADAS-2: a revised tool for the quality assessment of diagnostic accuracy studies. Ann Intern Med 2011;155:529–36. 22007046 10.7326/0003-4819-155-8-201110180-00009

[23] Deeks JJ Macaskill P Irwig L The performance of tests of publication bias and other sample size effects in systematic reviews of diagnostic test accuracy was assessed. J Clin Epidemiol 2005;58:882–93. 16085191 10.1016/j.jclinepi.2005.01.016

